# Automated in situ chromatin profiling efficiently resolves cell types and gene regulatory programs

**DOI:** 10.1186/s13072-018-0243-8

**Published:** 2018-12-21

**Authors:** Derek H. Janssens, Steven J. Wu, Jay F. Sarthy, Michael P. Meers, Carrie H. Myers, James M. Olson, Kami Ahmad, Steven Henikoff

**Affiliations:** 1 Basic Sciences Division, Fred Hutchinson Cancer Research Center, 1100 N. Fairview Ave, Seattle, WA 98109 USA; 2 Molecular Engineering and Sciences Institute, University of Washington, Seattle, WA 98195 USA; 3 Cancer and Blood Disorder Center, Seattle Children’s Hospital, 4800 Sand Point Way, Seattle, WA 98105 USA; 4 Clinical Research Division, Fred Hutchinson Cancer Research Center, 1100 N. Fairview Ave, Seattle, WA 98109 USA; 5 Howard Hughes Medical Institute, Chevy Chase, MD USA

**Keywords:** CUT&RUN, Histone modifications, Transcription factors, Chromatin regulators

## Abstract

**Background:**

Our understanding of eukaryotic gene regulation is limited by the complexity of protein–DNA interactions that comprise the chromatin landscape and by inefficient methods for characterizing these interactions. We recently introduced CUT&RUN, an antibody-targeted nuclease cleavage method that profiles DNA-binding proteins, histones and chromatin-modifying proteins in situ with exceptional sensitivity and resolution.

**Results:**

Here, we describe an automated CUT&RUN platform and apply it to characterize the chromatin landscapes of human cells. We find that automated CUT&RUN profiles of histone modifications crisply demarcate active and repressed chromatin regions, and we develop a continuous metric to identify cell-type-specific promoter and enhancer activities. We test the ability of automated CUT&RUN to profile frozen tumor samples and find that our method readily distinguishes two pediatric glioma xenografts by their subtype-specific gene expression programs.

**Conclusions:**

The easy, cost-effective workflow makes automated CUT&RUN an attractive tool for high-throughput characterization of cell types and patient samples.

**Electronic supplementary material:**

The online version of this article (10.1186/s13072-018-0243-8) contains supplementary material, which is available to authorized users.

## Background

Cells establish their distinct identities by altering activity of the *cis*-regulatory DNA elements that control gene expression [1, 2]. Promoter elements lie near the 5′ transcriptional start sites (TSSs) of all genes, whereas distal *cis*-regulatory elements such as enhancers often bridge long stretches in the DNA to interact with select promoters and direct cell-type-specific gene expression [1, 2]. Defects in the nuclear proteins that recognize these *cis*-regulatory elements underlie many human diseases that often manifest in specific tissues and cell types [3–7]. However, we are only just beginning to appreciate how assessing the activity of *cis*-regulatory elements may be used in clinical settings for patient diagnosis [8]. To provide a reference for molecular diagnosis of patient samples, efforts are underway to generate a comprehensive atlas of cells in the human body [9, 10]. Characterizing cell-type-specific chromatin landscapes is essential for this atlas; however, technical limitations have prevented implementation of traditional approaches for genome-wide profiling of chromatin proteins on the scales necessary for this project.

Despite the growing awareness that epigenetic derangements underlie many human diseases [11], very few methods for high-throughput profiling of epigenomic information are available. Realizing the clinical potential of epigenomic technologies requires robust, scalable approaches that can profile large numbers of patient samples in parallel. Chromatin immunoprecipitation with antigen-specific antibodies combined with massively parallel sequencing (ChIP-seq) has been used extensively for epigenome profiling, but this method is labor-intensive, prone to artifacts [12–14], and requires high sequencing depth to distinguish weak signals from genomic background noise. Although semi-automated implementations of ChIP-seq exist, these begin with cross-linking cells and solubilization by sonication [15–17], steps that are difficult to scale and to control for reproducibility. The combination of these factors has prevented implementation of ChIP-seq in clinical laboratory settings. Recently, we have introduced CUT&RUN as an alternative chromatin profiling technique that uses factor-specific antibodies to tether micrococcal nuclease (MNase) to genomic binding sites [18, 19]. The targeted nuclease cleaves chromatin around the binding sites, and the released DNA is sequenced using standard library preparation techniques, resulting in efficient mapping of protein-DNA interactions. CUT&RUN has very low backgrounds, which greatly reduces sample amounts and sequencing costs required to obtain high-quality genome-wide profiles [18, 20].

Here, we modify the CUT&RUN protocol to profile chromatin proteins and modifications in a 96-well format on a liquid handling robot, beginning with permeabilized cells and ending with barcoded libraries that are ready to be pooled for sequencing. By applying this method to the H1 human embryonic stem cell (hESC) line and the K562 leukemia cell line, we demonstrate that AutoCUT&RUN can be used to identify cell-type-specific promoter and enhancer activities, providing a means to quantitatively distinguish cell-types based on their unique gene regulatory programs. In addition, we show that this method is able to define chromatin features from frozen solid tumor samples, setting the stage to analyze typical clinical specimens at low cost. AutoCUT&RUN is ideal for high-throughput studies of chromatin-based gene regulation, allowing for examination of chromatin landscapes in patient samples and expanding the toolbox for epigenetic medicine.

## Results

### An automated platform for genome-wide profiling of chromatin proteins

**Fig. 1:**
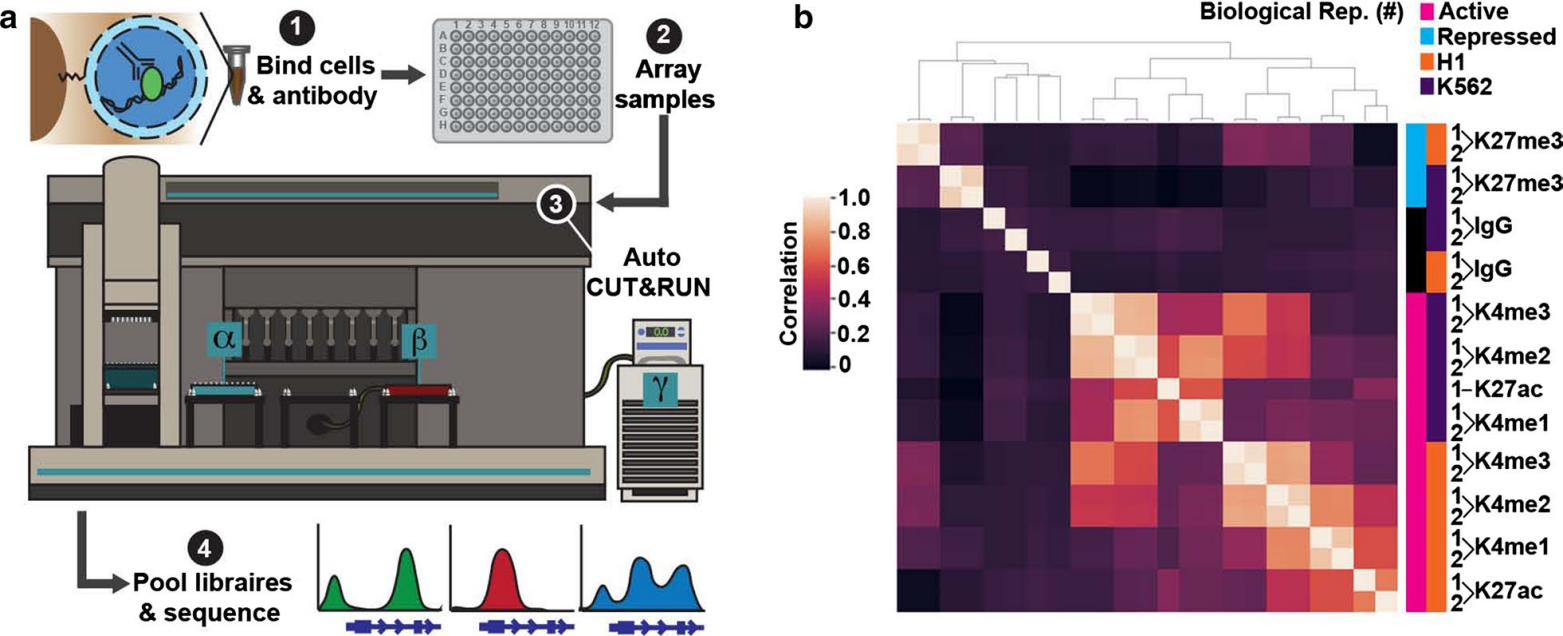
An automated platform for high-throughput in situ profiling of chromatin proteins. **a** AutoCUT&RUN workflow. (1) Cells or tissue are bound to concanavalin A-coated beads, permeabilized with digitonin, and incubated with an antibody targeting a chromatin protein. (2) Samples are arrayed in a 96-well plate and (3) processed on a Biomek robot fitted with a 96-well magnetic plate for magnetic separation during washes (α), and an aluminum chiller block (β) routed to a circulating water bath (γ) for temperature control. (4) AutoCUT&RUN produces in 2 days up to 96 libraries that are ready to be pooled and sequenced. **b** Hierarchically clustered correlation matrix of AutoCUT&RUN profiles of histone-H3 modifications that mark active (pink) and repressed (blue) chromatin in H1 (orange) and K562 (purple) cells. Pearson correlations were calculated using the log_2_-transformed values of read counts split into 500 bp bins across the genome

To adapt CUT&RUN to an automated format we equipped a Beckman Biomek FX liquid handling robot for magnetic separation and temperature control (Fig. 1a). First, cells are bound to concanavalin A-coated magnetic beads, allowing all subsequent washes to be performed by magnetic separation. Bead-bound samples are then incubated with antibodies, and up to 96 samples are arrayed in a plate (Fig. 1a). Successive washes, tethering of a proteinA-MNase fusion protein, cleavage of DNA, and release of cleaved chromatin fragments into the sample supernatant are performed on the Biomek (Additional file 1: Fig. S1a). A major stumbling block to automating epigenomics protocols is that they typically require purification of small amounts of DNA prior to library preparation. To overcome this hurdle, we developed a method to polish the DNA ends in chromatin fragments for direct ligation of Illumina library adapters (Additional file 1: Fig. S1a). End-polishing and adapter ligation are performed on a separate thermocycler, and deproteinated CUT&RUN libraries are purified on the Biomek using Ampure XP magnetic beads both before and after PCR enrichment. This AutoCUT&RUN protocol allows a single operator to generate up to 96 libraries in 2 days that are ready to be pooled and sequenced (Fig. 1a) (https://www.protocols.io/view/autocut-run-genome-wide-profiling-of-chromatin-pro-ufeetje).

To test the consistency of AutoCUT&RUN, we simultaneously profiled two biological replicates of H1 hESCs and K562 cells using antibodies targeting four histone modifications that mark active chromatin states (H3K4me1, H3K4me2, H3K4me3, and H3K27ac) and one repressive modification (H3K27me3). Comparing the global distribution of reads for each histone mark, we found that samples highly correlate with their biological replicate and cluster together in an unbiased hierarchical matrix (Fig. 1b). Additionally, the genome-wide profiles of the four active histone marks clustered together within a given cell type and separated away from the repressive histone mark H3K27me3 (Fig. 1b). These profiles represent antibody-specific signals, as all five are poorly correlated with an IgG-negative control. Together, these results indicate that AutoCUT&RUN chromatin profiling reproducibly captures the cell-type-specific distributions of histone marks.

In addition to profiling histone modifications, we also examined whether AutoCUT&RUN can be applied to mapping DNA-binding transcription factors. We tested the performance of AutoCUT&RUN with two transcription factors, the histone locus-specific gene regulator NPAT, and the insulator protein CTCF [21, 22]. AutoCUT&RUN profiles of both NPAT and CTCF are highly specific for their expected targets in both H1 and K562 cells (Additional file 1: Fig. S1b, c). Thus, AutoCUT&RUN is suitable for high-throughput, genome-wide profiling of diverse DNA-binding proteins.

### Comparison of AutoCUT&RUN to ChIP-seq

**Fig. 2:**
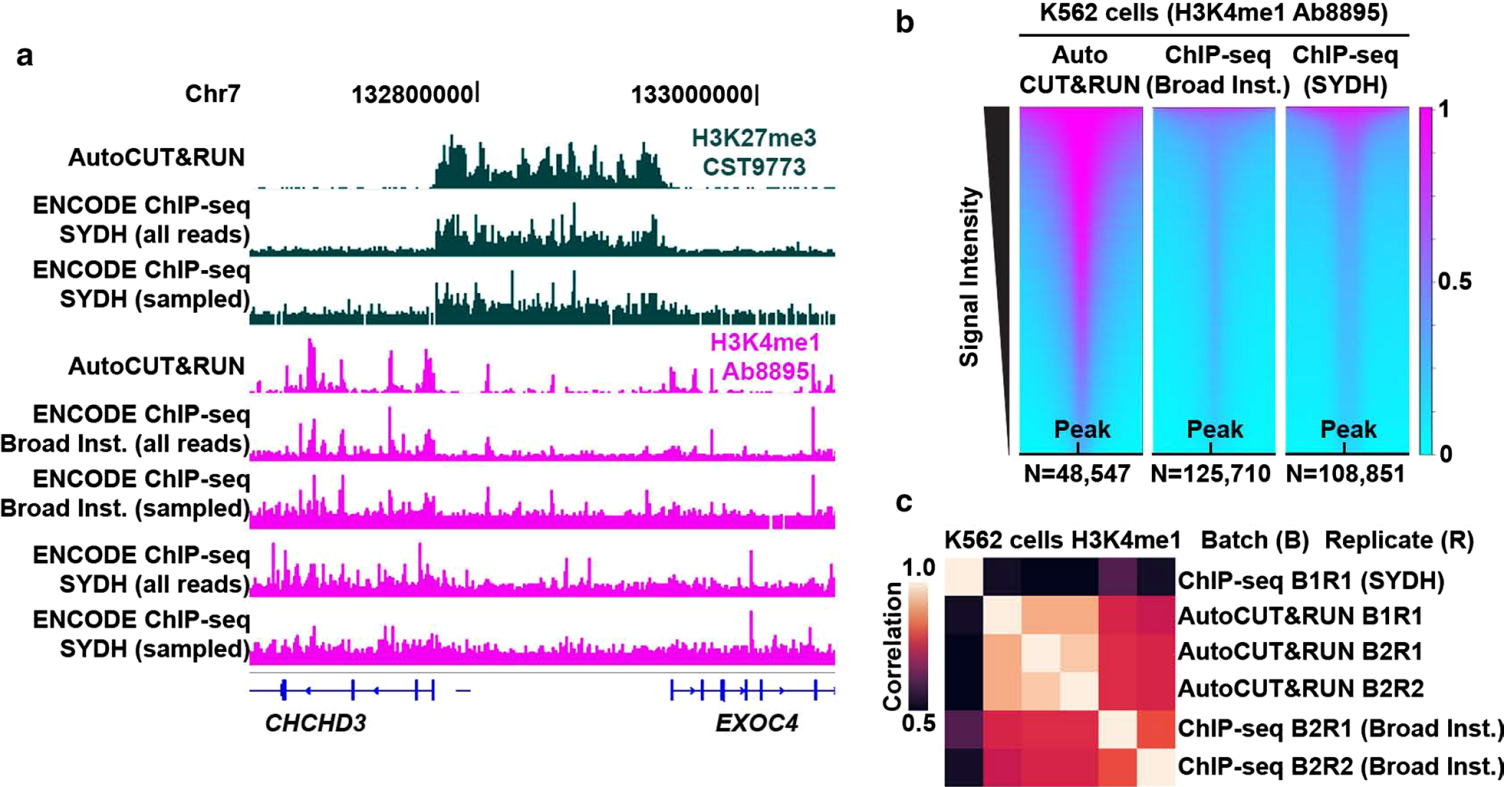
Comparison of AutoCUT&RUN versus ENCODE ChIP-seq. **a** A representative silenced domain flanked by active genes is shown for human K562 cells probed using the same antibodies by either AutoCUT&RUN or ChIP-seq. For each comparison, ChIP-seq tracks are shown that include either all sequenced fragments or the same number of sampled fragments as the AutoCUT&RUN data. **b** Heat map comparison of H3K4me1 Ab8895 ± 1 kb around peak centers (summits) called by MACS2 for AutoCUT&RUN data and for ENCODE ChIP-seq data (broad peaks by Broad Institute and narrow peaks by SYDH). Signal intensities reflect the relative number of reads that fall within peaks. **c** Correlation matrix comparing batches (*B*) and replicates (*R*) between AutoCUT&RUN and ENCODE ChIP-seq datasets for H3K4me1 Ab8895

We previously showed that the low backgrounds and high efficiency of CUT&RUN allowed for much lower DNA sequencing read depths than required for conventional ChIP-seq to obtain good feature definition. To determine whether improved performance relative to ChIP-seq extends to AutoCUT&RUN, we first identified the histone modification datasets from the ENCODE project that used the same antibodies and manufacturer catalog numbers as we used. A representative region is shown for direct comparison of tracks between AutoCUT&RUN and ENCODE (Fig. 2a). In all comparisons, the ENCODE datasets are seen to be much noisier than the CUT&RUN datasets, despite the fact that there were ~ 2 to 3 times as many mapped reads in the ENCODE datasets, pooling all of the reads from the two replicates available in the Gene Expression Omnibus (GEO). The requirement for much deeper sequencing using ChIP-seq relative to CUT&RUN is illustrated by the effect of downsampling the ENCODE datasets to a number of reads equivalent to the number of CUT&RUN fragments, where in the case of H3K4me1, the feature definition from ChIP-seq becomes dramatically reduced, whereas the same number of mapped CUT&RUN fragments shows clear peaks with much lower background than seen for either the Broad Institute or SYDH ENCODE tracks for all comparisons. We confirmed that the higher data quality of CUT&RUN extends to genome-wide analysis, where heat maps of MACS2 peak calls for CUT&RUN show much better signal-to-noise than heat maps for corresponding ENCODE datasets using ENCODE-generated peak calls (Fig. 2b).

The fixation, sonication, and immunoprecipitation steps of ChIP-seq have the potential to introduce significant batch-effect variability between experiments [23, 24], often making it difficult to directly compare large ChIP-seq datasets generated by different laboratories. To examine whether AutoCUT&RUN reduces this batch-effect variability, we compared the global distribution of reads for H3K4me1 in K562 cells generated from multiple different AutoCUT&RUN and ENCODE ChIP-seq experiments. This analysis revealed that biological replicates profiled by AutoCUT&RUN in different batches have a similar correlation with biological replicates profiled in parallel, indicating there is very little batch-effect variability between AutoCUT&RUN experiments (Fig. 2c). Furthermore, the correlation between AutoCUT&RUN samples and the Broad Institute ChIP-seq samples was similar to the correlation of Broad Institute ChIP-seq replicates with each other (Fig. 2c). In agreement with the visual comparison of tracks (Fig. 2a), this demonstrates the genome-wide profiles of H3K4me1 generated by AutoCUT&RUN are consistent with results obtained using ChIP-seq. However, the correlation between the Broad Institute ChIP-seq replicates and the SYDH ChIP-seq replicate was far lower than that observed between different AutoCUT&RUN batches, reaffirming the inherent difficulty in reproducing ChIP-seq results (Fig. 2c). We conclude that by eliminating many of the potential sources of batch-effects associated with ChIP-seq, AutoCUT&RUN significantly improves reproducibility between experiments, which will facilitate the adaptation for clinical applications.

**Fig. 3:**
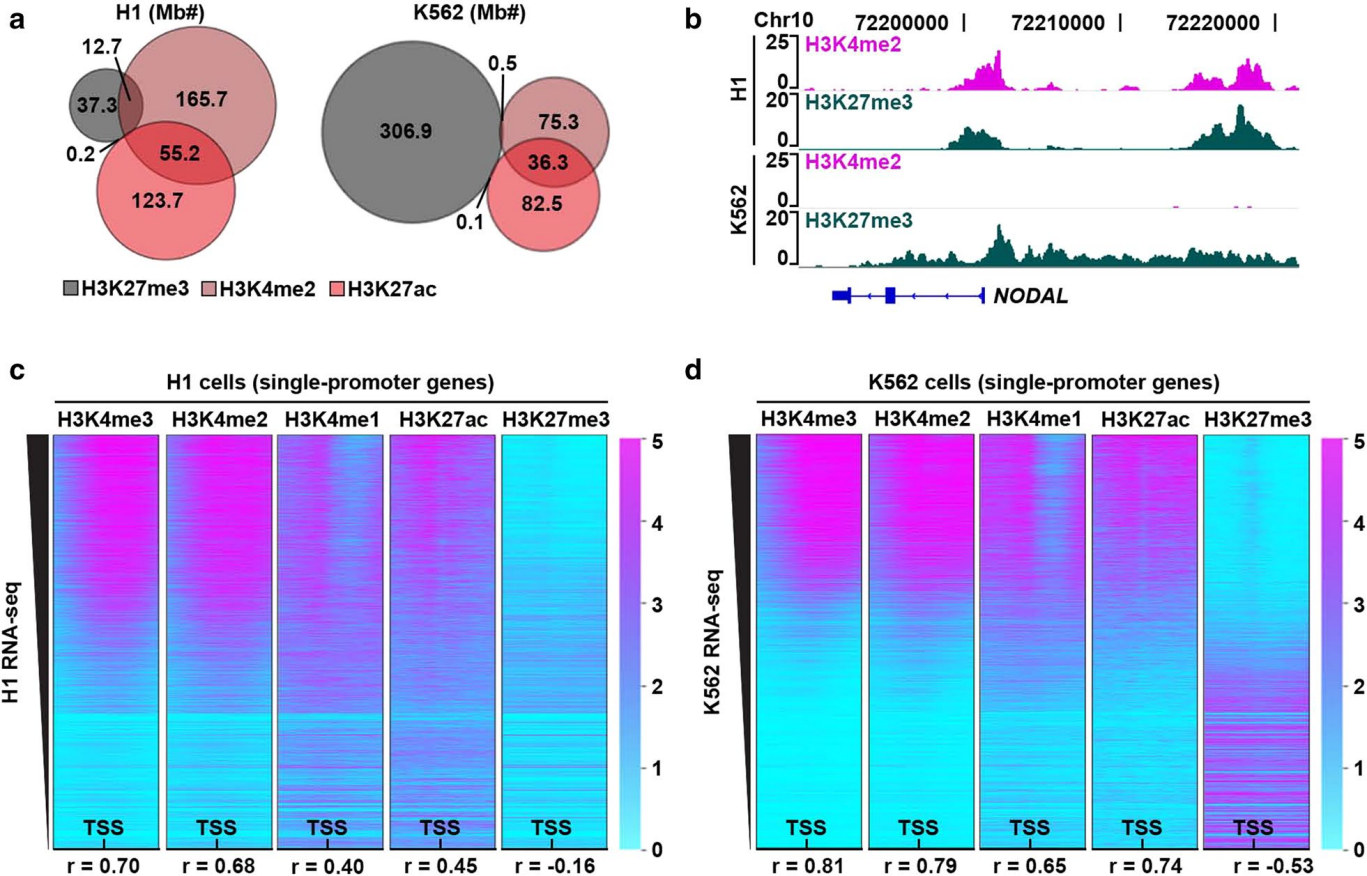
AutoCUT&RUN reproduces the expected chromatin landscape of H1 and K562 cells. **a** Scaled Venn diagrams showing the relative amount of the genome that falls within H3K27me3 (gray), H3K4me2 (brown), and H3K27ac (red) domains in H1 cells and K562 cells. Numbers indicate megabases (Mb). **b** Genome browser tracks showing the overlap of H3K4me2 and H3K27me3 in H1 cells, as well as the expansion of H3K27me3 domains and loss of overlap with H3K4me2 in K562 cells at a representative locus (*NODAL*). **c** Heat maps showing the distribution of AutoCUT&RUN profiles of histone modifications in H1 cells centered on the TSSs of genes with a single promoter, oriented left-to-right according to the 5′-to-3′ direction of transcription and rank-ordering according to RNA-seq values (FPKM). **d** Heat maps showing the distribution of AutoCUT&RUN histone modification profiles on transcriptionally active and repressed promoters in K562 cells. Pearson correlations (*r* value) between AutoCUT&RUN profiles of individual histone marks around these TSSs and their corresponding RNA-seq values are indicated

We next examined whether AutoCUT&RUN profiles recapitulate global chromatin features that have been previously ascribed to hESCs using ChIP-seq. To maintain their developmental plasticity, hESCs are thought to have a generally “open,” hyper-acetylated chromatin landscape interspersed with repressed domains of “bivalent” chromatin, marked by overlapping H3K27me3 and H3K4 methylation [25–28]. AutoCUT&RUN recapitulates these features of hESCs; we observed that H1 cells have increased H3K27ac as compared to the lineage-restricted K562 cell line, whereas domains of the repressive histone mark H3K27me3 are rare in H1 cells, but prevalent in K562 cells (Fig. 3a). We also observed extensive overlap between H3K27me3 and H3K4me2 signals in H1 cells, but not K562 cells (Fig. 3a, b). Thus, AutoCUT&RUN profiles are consistent with the specialized chromatin features found in hESCs using ChIP-seq.

Post-translational modifications to the H3 histone tail closely correlate with transcriptional activity [29]. To determine whether our AutoCUT&RUN profiles of histone modifications are indicative of transcriptional activity, we examined the distribution of the five histone marks around the transcriptional start sites (TSSs) of genes, rank-ordered according to RNA-seq expression data (Fig. 3c, d) [30]. We find the active mark H3K4me3 is the most highly correlated with expression in both cell types (*r* = 0.70 and 0.81 for H1 and K562, respectively), followed by H3K4me2 and H3K27ac (Fig. 3c, d). The repressive histone mark H3K27me3 is anti-correlated with expression (*r* = −0.16 and −0.53 in H1 and K562, respectively) (Fig. 3c, d). We conclude AutoCUT&RUN for these histone marks provides a strategy to identify cell-type-specific gene regulatory programs.

### Modeling cell-type-specific gene expression from AutoCUT&RUN profiles

**Fig. 4:**
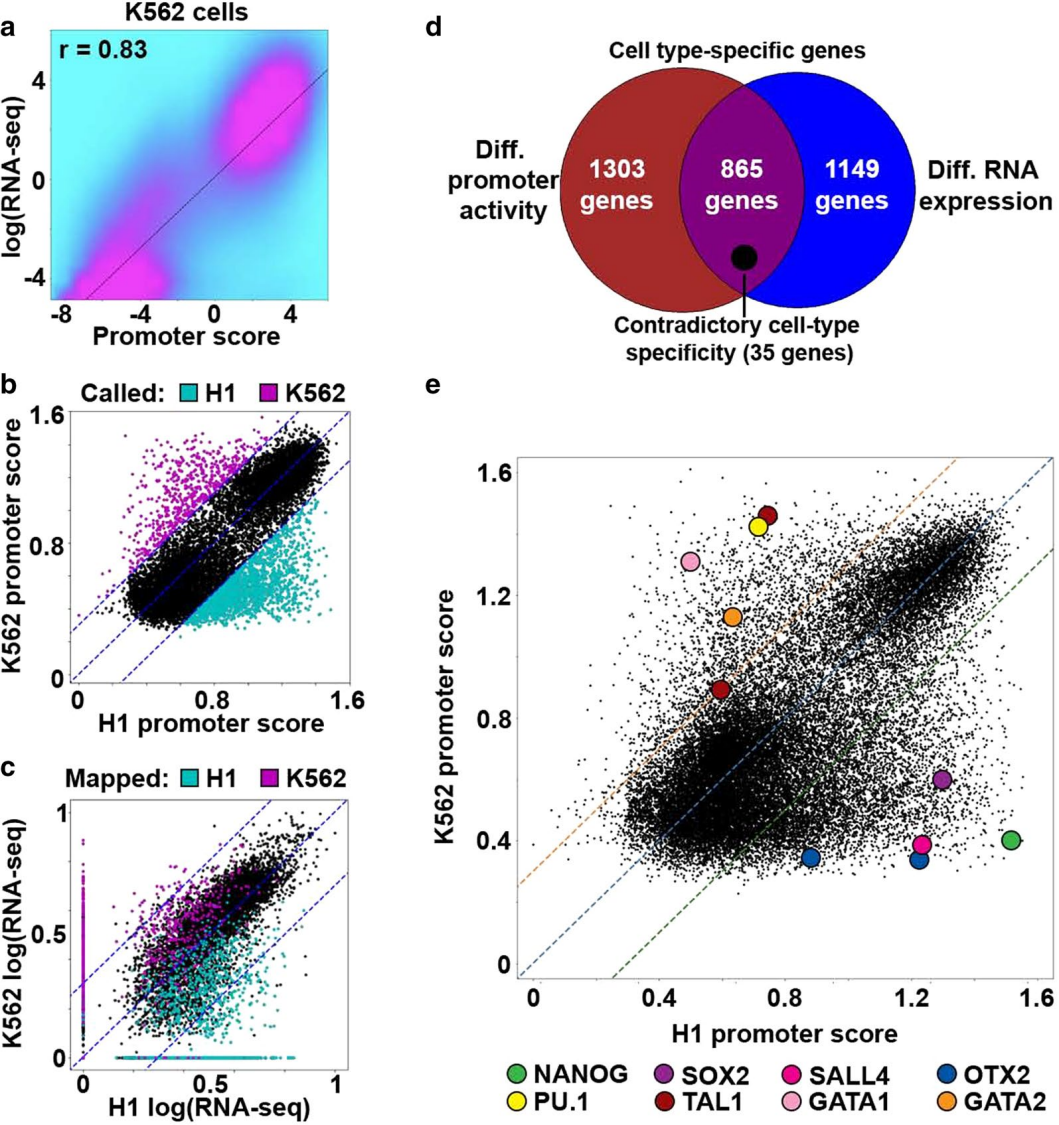
A linear regression model accurately predicts cell-type-specific promoter activity. **a** Density scatterplot comparing RNA-seq values for single-promoter genes to K562 promoter scores predicted by the model trained on K562 data. **b** Scatterplot of promoter chromatin scores for single-promoter genes in H1 and K562 cells. Colored dots indicate that the promoter scores are ≥ twofold enriched in either H1 cells (cyan) or K562 cells (magenta). **c** Scatterplot of promoter scores that are ≥ twofold enriched in either H1 cells (cyan) or K562 cells (magenta) mapped onto their corresponding RNA-seq values. Blue dotted lines indicate the twofold difference cutoff. **d** Scaled Venn diagram showing the overlap between genes called as cell type specific according to their promoter scores, or according to their RNA expression values. Genes predicted to have contradictory cell type specificities according to promoter activity modeling versus RNA-seq are indicated (scaled black circle). **e** Scatterplot comparing the H1 and K562 scores of all promoters separated by ≥ 2 kb. Master regulators of H1 and K562 cell identities are indicated as colored circles. Both *OTX2* and *TAL1* have two promoters that can be distinguished

To use AutoCUT&RUN data to compare cell types and distinguish their gene regulatory programs, we wanted to develop a continuous metric that incorporates both active and repressive chromatin marks. RNA-seq has been widely used to identify cell-type-specific gene expression programs [30], so we used RNA-seq data as a reference for training a weighted linear regression model that incorporates normalized H3K4me2, H3K27ac, and H3K27me3 read counts to assign promoters a relative activity score. We initially focused our analysis on genes with a single TSS that could be unambiguously assigned RNA-seq values. H3K4me2 was selected over H3K4me3 and H3K4me1 because H3K4me2 is uniquely applicable for modeling the activity of both proximal and distal *cis*-regulatory elements (see below). When applied to K562 cells, promoter chromatin scores correlate very well with RNA-seq values (*r* = 0.83) (Fig. 4a), providing a comparable power for predicting gene expression as similar models that used up to 39 histone modifications mapped by ChIP-seq (*r* = 0.81) [29]. In addition, our weighted model trained on K562 cells performs well when applied to H1 cells (Additional file 1: Fig. S2a, b), indicating that the linear model and data quality are sufficiently robust to assign promoter scores to diverse cell types.

Next, we examined whether AutoCUT&RUN accurately identifies promoters with cell-type-specific activity. By calling promoter scores that were enriched more than twofold in either H1 or K562 cells, we identified 2168 cell-type-specific genes and approximately 40% of these genes (865) were also differentially enriched between H1 and K562 cells according to RNA-seq (Fig. 4b–d). However, promoter activity modeling did not capture transcriptional differences for 1149 genes (Fig. 4d, Additional file 1: Fig. S2c, d), implying that these genes are differentially expressed without changes in the chromatin features included in our model. This differential sensitivity between methods suggests the three histone marks included in our chromatin model may more accurately predict the cell-type-specific expression of certain classes of genes than others. Indeed, we find the 865 cell-type-specific genes identified by both promoter activity modeling and RNA-seq are highly enriched for developmental regulators, whereas the genes called by either promoter scores or RNA-seq alone are not nearly as enriched for developmental GO terms (Fig. 4d, Additional file 1: Fig. S2e–g, Additional file 2: Table S1). In addition, only 35 genes display contradictory cell-type specificities according to promoter chromatin scores and RNA-seq (Fig. 4d). This demonstrates AutoCUT&RUN profiling of these widely studied modifications to the H3 histone tail can be applied to accurately distinguish between cell-type-specific developmental regulators.

To determine whether AutoCUT&RUN data recapitulate the expression of cell-type-specific transcription factors, we expanded our analysis to include all promoters. We find that components of the hESC pluripotency network (*NANOG*, *SOX2*, *SALL4*, and *OTX2*) have higher promoter chromatin scores in H1 cells, while regulators of hematopoietic progenitor cell fate (*PU.1*, *TAL1*, *GATA1*, and *GATA2*) are enriched in K562 cells (Fig. 4e, Additional file 2: Table S1) [31, 32]. This method also identifies activities of alternative promoters (e.g., at the *OTX2* and *TAL1* genes), providing an indication of the specific gene isoforms that are expressed in a given cell type (Fig. 4e). We conclude that AutoCUT&RUN can distinguish between master regulators of cellular identity, providing a powerful tool to characterize cell-types in a high-throughput format.

### Profiling tumors by AutoCUT&RUN

**Fig. 5:**
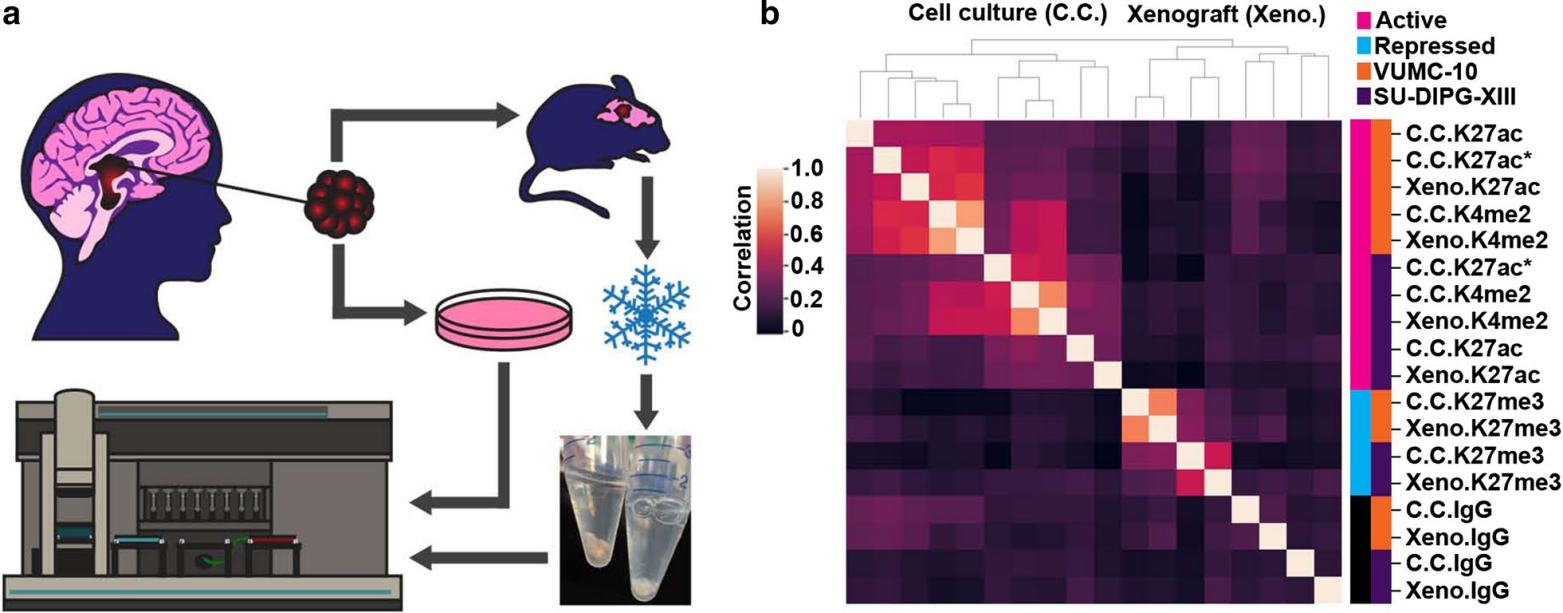
AutoCUT&RUN is suitable for profiling the chromatin landscape of frozen tumor samples. **a** DMG experimental setup. Two DMG cell lines derived from a similar region of the brainstem were grown as xenografts in the brains of immunocompromised mice, and upon forming tumors were resected and frozen. Xenografts were thawed and processed by AutoCUT&RUN in parallel with control DMG samples harvested directly from cell culture. **b** Hierarchically clustered correlation matrix of AutoCUT&RUN profiles of histone-H3 modifications that mark active (pink) and repressed (blue) chromatin in VUMC-10 (orange) and SU-DIPG-XIII (purple) cells grown in cell culture (C.C.) or as xenografts (Xeno.). As a quality control, H3K27ac was also profiled manually in these cell lines using a different antibody (*). Pearson correlations were calculated using the log_2_-transformed values of read counts split into 500 bp bins across the genome

Typical clinical samples often contain small amounts of material and have been flash-frozen, and although ChIP-seq has been applied to flash-frozen tissue samples, available methods are not sufficiently robust for diagnostic application. In addition, translational samples from xenografts, which are increasingly being used in clinical settings to probe treatment strategies for patients with high-risk malignancies [34]. These specimens can be extremely challenging to profile by ChIP-seq as they often contain a significant proportion of mouse tissue and so require extremely deep sequencing to distinguish signal from noise. To test whether AutoCUT&RUN is suitable for profiling frozen tumor specimens, we obtained two diffuse midline glioma (DMG) patient-derived cell lines (VUMC-10 and SU-DIPG-XIII) that were autopsied from similar regions of the brainstem, but differ in their oncogenic backgrounds [33]. SU-DIPG-XIII is derived from a tumor containing an H3.3K27M “oncohistone” mutation, which results in pathologically low levels of PRC2 activity, and because of this has been called an “epigenetic” malignancy. In contrast, VUMC-10 is a *MYCN*-amplified, histone wild-type brainstem glioma [34]. Both of these DMG cell lines readily form xenografts in murine models, and we applied AutoCUT&RUN to profile histone modifications in VUMC-10 and SU-DIPG-XIII xenografts that were seeded in the brains of mice and then resected upon tumor formation and frozen under typical clinical conditions (Fig. 5a). For comparison, on the same AutoCUT&RUN plate we profiled the parental DMG cell lines grown in culture (Fig. 5a). Again, we found that replicates were highly concordant, so we combined them for further analysis. Importantly, cell culture samples were highly correlated with the same mark profiled in the corresponding frozen xenografts, and AutoCUT&RUN on xenograft tissues and cell culture samples produced similar data quality (Fig. 5b, Additional file 1: Fig. S3). Thus, AutoCUT&RUN reliably generates genome-wide chromatin profiles from frozen tissue samples.

**Fig. 6:**
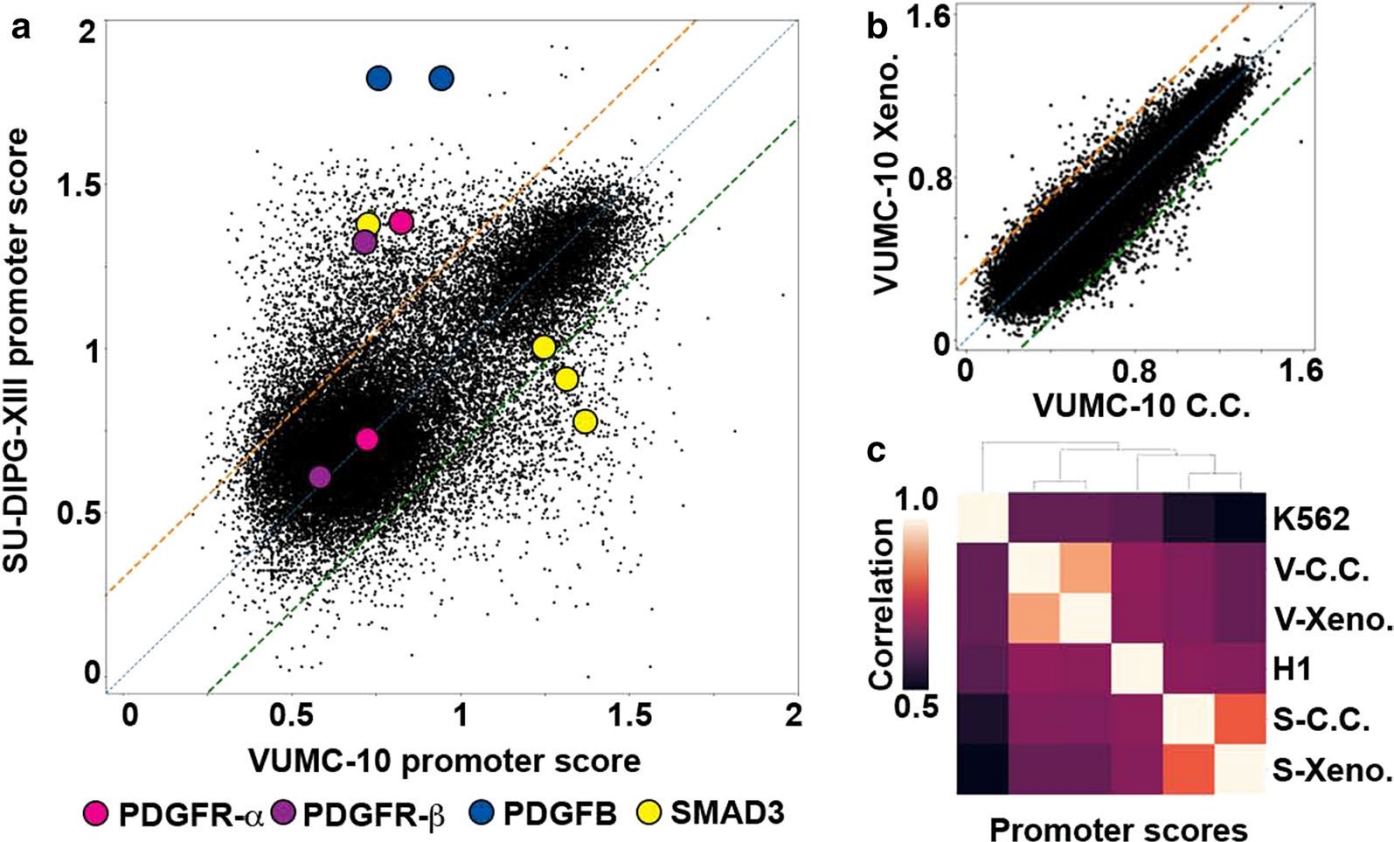
Promoter activity modeling distinguishes gene activities in DMG samples. **a** Scatterplot comparing the promoter scores of VUMC-10 and SU-DIPG-XIII cell culture samples. Locations of the promoters of several cell signaling components implicated in tumor growth are indicated as colored circles. **b** Scatterplot comparing the promoter scores of VUMC-10 cell culture (C.C.) and xenograft (Xeno.) samples. Only 388 promoters have a ≥ twofold difference in activity modeling scores between these samples. **c** Hierarchically clustered matrix of Spearman correlations of promoter chromatin scores between VUMC-10 (V) and SU-DIPG-XIII (S) cells grown in cell culture (C.C.) or as xenografts (Xeno.), as well as H1 and K562 cells

Stratification of patient malignancies is becoming increasingly dependent on molecular diagnostic methods that distinguish tumor subtypes derived from the same tissues. Our VUMC-10 and SU-DIPG-XIII samples provide an excellent opportunity to explore the potential of using AutoCUT&RUN to classify tumor specimens according to their subtype-specific regulatory elements. By applying promoter modeling to these samples, we identified 5006 promoters that show differential activity between VUMC-10 and SU-DIPG-XIII cells (Fig. 6a, Additional file 2: Table S1). Consistent with the glial origins of these tumors, both the VUMC-10- and SU-DIPG-XIII-specific promoters are significantly enriched for genes involved in nervous system development (Additional file 1: Fig. S4a, b). Genes involved in cell signaling are also overrepresented in SU-DIPG-XIII cells (Additional file 1: Fig. S4b); for example, the promoters of the *PDGFR* gene as well as its ligand *PDGF* are highly active in SU-DIPG-XIII cells (Fig. 6a). This is consistent with the observation that DMGs frequently contain activating mutations in PDGFR-α that promote tumor growth [5]. In addition, one promoter of the *SMAD3* gene, a component of the TGF-β signaling pathway [35], is specifically active in SU-DIPG-XIII cells, whereas two different *SMAD3* promoters are active in VUMC-10 cells (Fig. 6a, Additional file 1: Fig. S3). In comparison, our model indicates that only 388 promoters differ between VUMC-10 xenografts and cultured cells, and 1619 promoters differ between SU-DIPG-XIII samples (Fig. 6b, Additional file 1: Fig. S5c). In addition, comparing promoter chromatin scores in an unbiased correlation matrix also indicates DMG xenografts are far more similar to their corresponding cell culture samples than they are to other DMG subtypes or to H1 or K562 cells (Fig. 6c). This suggests that AutoCUT&RUN can be applied to identify promoters that display tumor subtype-specific activity, providing a reliable method to assign cellular identities to frozen tumor samples, as well as an indication of the signaling pathways that may be driving tumor growth and potential susceptibility to therapeutic agents.

### High-throughput mapping of cell-type-specific enhancers

**Fig. 7:**
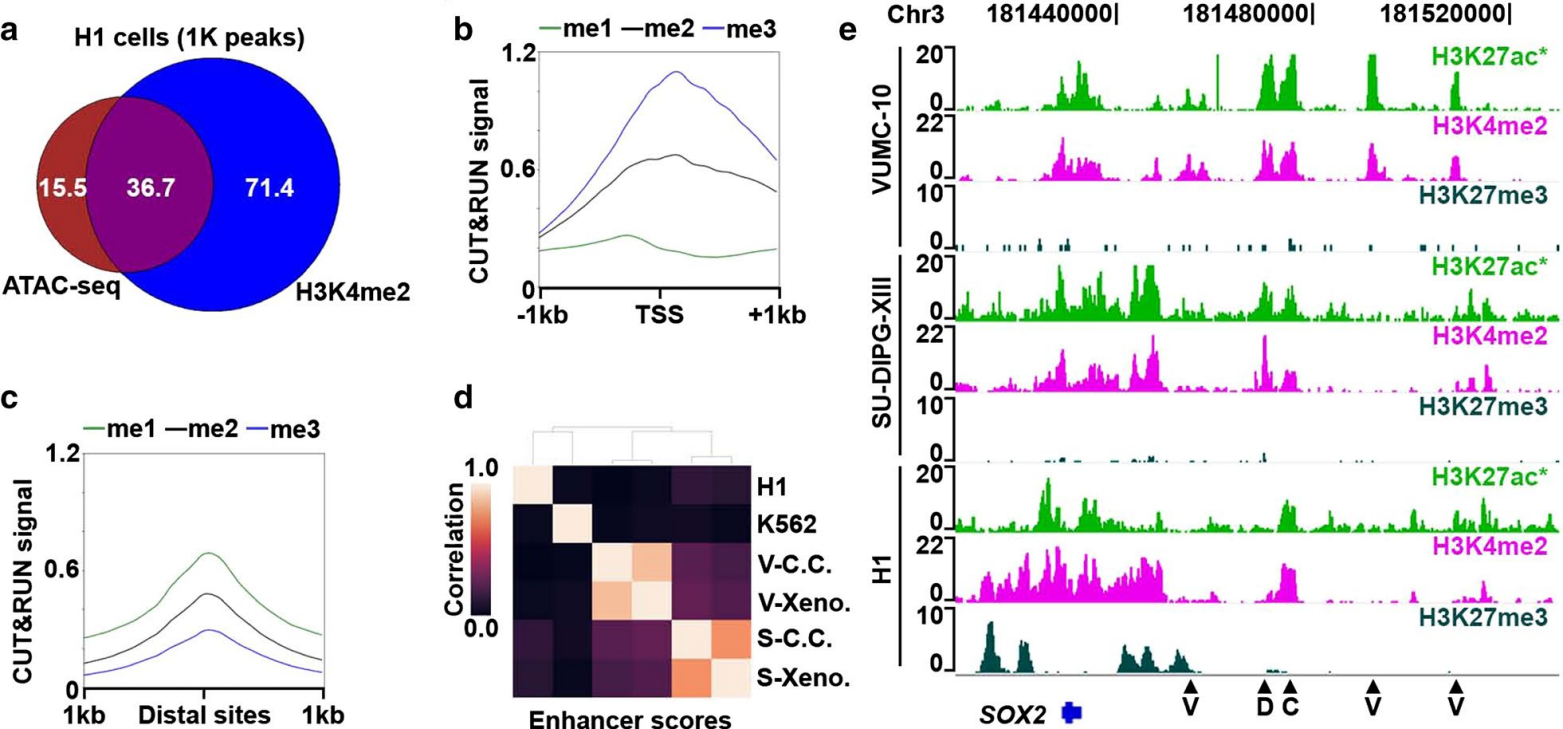
AutoCUT&RUN identifies cell-type-specific enhancer elements. **a** Scaled Venn diagram showing the overlap of accessible chromatin sites (ATAC-seq peaks) and peaks called on H3K4me2 AutoCUT&RUN profiles in H1 cells. Numbers are provided as 1 thousand peak units. **b** Mean enrichment of H3K4me1 (green) H3K4me2 (black) and H3K4me3 (blue) over all H3K4me2 +/ATAC + TSSs. **c** Mean enrichment of H3K4me1 (green) H3K4me2 (black) and H3K4me3 (blue) over all H3K4me2 +/ATAC + distal sites. **d** Hierarchically clustered matrix of Spearman correlations of enhancer chromatin scores in VUMC-10 (V) and SU-DIPG-XIII (S) cells grown in cell culture (C.C.) or as xenografts (Xeno.), as well as H1 and K562 cells. **e** Genome browser tracks showing the location of putative enhancer elements (arrow heads) that are specific to VUMC-10 cells (V), both DMG cell lines (D), or common to DMG cells and H1 cells (C) at a representative locus (*SOX2*). In VUMC-10 and SU-DIPG-XIII cells, the H3K27ac track that is shown was also profiled manually as a quality control (*)

The cell-type-specific activities of gene promoters are often established by incorporating signals from distal *cis*-regulatory elements, such as enhancers [1, 2]. Similar to promoters, enhancers also display H3K4me2 [36], and active enhancers are typically marked by H3K27ac, whereas repressed enhancers are marked by H3K27me3 [28, 37, 38]. Therefore, we reasoned that the AutoCUT&RUN profiles we used to model promoter activity should also allow identification of cell-type-specific enhancers. To investigate this possibility, we first compared our H1 data to available chromatin accessibility maps generated by ATAC-seq, which are enriched for both active promoters and enhancers [39, 40]. Of the marks we profiled, we find H3K4me2 peaks show the highest overlap with ATAC-seq (Fig. 7a, Additional file 1: Fig. S5a), and identify 36,725/52,270 ATAC-seq peaks (~ 70%). Interestingly, H3K4me2 defines an additional 71,397 peaks that were not called by ATAC-seq (Fig. 7a, Additional file 1: Fig. S5a). Many of these H3K4me2-specific peaks show a low, but detectable ATAC-seq signal (Additional file 1: Fig. S5b), indicating they may correspond to repressed promoters and enhancers. Consistent with this interpretation, on average H3K4me2+/ATAC-TSSs have higher H3K27me3 signals than H3K4me2+/ATAC+ TSSs (Additional file 1: Fig. S5c). H3K4me2+/ATAC+ peaks that overlap with annotated TSSs are enriched for H3K4me3, while those peaks that do not overlap TSSs are enriched for H3K4me1 (Fig. 7b, c, Additional file 1: Fig. S5d), suggesting that many of these distal peaks are enhancers [28, 41]. Thus, mapping sites of H3K4me2 by AutoCUT&RUN provides a sensitive method for defining the repertoire of active *cis*-regulatory elements that control gene expression programs.

Finally, we examined whether AutoCUT&RUN can be used to identify cell-type-specific enhancers. To expand the number of putative enhancer sites, we compiled a list of non-TSS peaks called on H3K4me2 profiles from all six cell lines and xenograft samples. Using our linear regression model, we then assigned these elements chromatin scores and examined their correlations between different cell types. We find that the chromatin scores of DMG cell culture samples and xenografts are highly correlated (*r* = 0.75 and 0.87 for SU-DIPG-XIII and VUMC-10 samples, respectively) (Fig. 7d). In contrast, the chromatin scores of SU-DIPG-XIII cells show a weak positive correlation with VUMC-10 cells (e.g. *r* = 0.19), indicating tumor subtype-specific differences. For example, different enhancers near the *SOX2* pluripotency gene are active in VUMC-10 cells than SU-DIPG-XIII or H1 cells (Fig. 7e), indicating that SU-DIPG-XIII cells resemble a more primitive neural stem cell type than VUMC-10 cells, as has been previously suggested [42]. Thus, modeling enhancer activity from AutoCUT&RUN profiles of chromatin marks is a highly discriminative method for stratifying cell types and tissue samples to inform patient diagnosis.

## Discussion

We adapted the CUT&RUN technique to an automated platform by developing direct ligation of chromatin fragments for Illumina library preparation, and implementing magnetic separation for the wash steps and library purification. AutoCUT&RUN generates 96 genome-wide profiles of antibody-targeted chromatin proteins in just 2 days, dramatically increasing the throughput and potential scale of studies to interrogate the chromatin landscape at a fraction of the cost of comparable lower-throughput methods.

A looming issue in the field of genomics is that extracting meaningful biological insights and clinical information from large datasets is often confounded by batch-effect variability that can arise from numerous sources including different experimental times, reagents, and operators [23, 24]. Automated versions of ChIP-seq have been described in which cross-linked and sonicated chromatin is immunoprecipitated on beads for automated library preparation, yielding results that are comparable to manual versions of the same or similar protocol [15–17]. However, cross-linking and sonication are the most difficult steps of a ChIP-seq pipeline to control, and so the non-automated steps of automated ChIP-seq represent a barrier to routine clinical application, where reproducibility is paramount. Moreover, the stark differences in quality between two different laboratories following the same ENCODE protocols using the same H3K4me1 antibody (Fig. 2) and subject to the extremely high standards imposed on the ENCODE consortium [43] illustrate how difficult it is to obtain uniform data quality in high-throughput ChIP-seq operations. In contrast, AutoCUT&RUN automates the entire process beginning with permeabilized cells or triturated tissues, and returns consistent data that have much better feature definition than that produced by ChIP-seq.

The low backgrounds and high efficiency inherent to antibody-targeted in situ profiling greatly reduce sequencing costs relative to ChIP-seq, surmounting the second major barrier to adoption of genome-wide epigenomic profiling for clinical applications. For example, we estimate that the cost per sample of the datasets we generated for this project was ~ $75 and required 2 days of technician time for 96 samples, ~ 1/10th the cost of commercial whole-exome sequencing (e.g., https://www.abmgood.com/Exome-Sequencing-Service.html). We expect that implementation as a routine service will allow institutional facilities to integrate AutoCUT&RUN into their sequencing pipelines for users who would provide only the cells or tissues and antibodies.

Using CUT&RUN, we have shown that profiling just three histone modifications (H3K27ac, H3K27me3, and H3K4me2) is sufficient to determine the cell-type-specific activities of developmentally regulated promoters and enhancers, providing a powerful quantitative metric to compare the epigenetic regulation of different cell types. This summary metric of chromatin features could be used to assess new cell types and tissue samples and to place them within a reference map of both healthy and diseased cell types. The automated workflow reduces technical and batch-to-batch variability between experiments, generating consistent profiles from biological replicates and from different sample types.

To continue optimizing AutoCUT&RUN, one could envision hardware modifications and computational development. By screening various antibody collections, the repertoire of nuclear proteins that can be efficiently profiled using AutoCUT&RUN would expand dramatically. In addition, the current AutoCUT&RUN protocol is optimized for a popular liquid handling robot, but a custom robot incorporating a reversibly magnetic thermocycler block would allow the CUT&RUN reaction and library preparation to be carried out in place, streamlining the protocol even further. Finally, metrics distinguishing cell types could be improved by incorporating additional aspects of the data, such as using a combination of both enhancer and promoter activities.

## Conclusions

The excellent reproducibility of profiling frozen tissue samples by AutoCUT&RUN has the potential to transform the field of epigenomic medicine [11]. Compared to other genomics approaches that are currently used for patient diagnosis, AutoCUT&RUN has the unique capacity to efficiently profile pathological chromatin proteins within diseased cells. For example, cancers caused by oncogenic chromatin-associated fusion proteins could be profiled by AutoCUT&RUN to provide a molecular diagnosis based on their chromatin landscapes, while simultaneously mapping the loci that are disrupted by the mutant fusion protein. This could provide a powerful tool for patient stratification, as well as a direct read-out of whether chromatin-modulating therapies such as histone deacetylase or histone methyltransferase inhibitors are having their intended effects.

## Methods

### AutoCUT&RUN

In conjunction with this work, a detailed AutoCUT&RUN protocol has been made publicly available on Protocols.io (https://www.protocols.io/view/autocut-run-genome-wide-profiling-of-chromatin-pro-ufeetje). Briefly, cells or tissue samples are bound to concanavalin A-coated magnetic beads (Bangs Laboratories, ca. no. BP531), permeabilized with digitonin, and bound with a protein specific antibody as previously described [18]. Samples are then arrayed in a 96-well plate and processed on a Beckman Biomek FX liquid handling robot equipped with a 96S Super Magnet Plate (Alpaqua SKU A001322) for magnetic separation of samples during wash steps, and an Aluminum Heat Block Insert for PCR Plates (V&P Scientific, Inc. VP741I6A) routed to a cooling unit to perform the MNase digestion reaction at 0–4 °C after the addition of 2 mM CaCl_2_. MNase digestion reactions are then stopped after 9 min by adding EGTA, which allows Mg^2+^ addition for subsequent enzymatic reactions. This step is critical for automation because it circumvents the need for DNA purification prior to library preparation. Chromatin fragments released into the supernatant during digestion are then used as the substrate for end-repair and ligation with barcoded Y-adapters. Prior to ligation, the A-tailing step is performed at 58 °C to preserve sub-nucleosomal fragments in the library [44, 45]. End-repair and adapter ligation reactions were performed on a separate thermocycler. Chromatin proteins are then digested with Proteinase K, and adapter ligated DNA fragments are purified on the Biomek FX using two rounds of pre-PCR Ampure bead cleanups with size selection. PCR enrichment reactions are performed on a thermocycler using the KAPA PCR kit (KAPA Cat#KK2502). Two rounds post-PCR Ampure bead cleanups with size selection are performed on the Biomek FX to remove unwanted proteins and self-ligated adapters.

The size distributions of AutoCUT&RUN libraries were analyzed on an Agilent 4200 TapeStation, and library yield was quantified by Qubit Fluorometer (Thermo Fisher). Up to 24 barcoded AutoCUT&RUN libraries were pooled per lane at equimolar concentration for paired-end 25 × 25 bp sequencing on a 2-lane flow cell on the Illumina HiSeq 2500 platform at the Fred Hutchinson Cancer Research Center Genomics Shared Resource.

### Antibodies

We used Rabbit anti-CTCF (1:100, Millipore Cat#07-729), Rabbit anti-NPAT (1:100, Termo Fisher Cat#PA5-66839), Rabbit anti-H3K4me1 (1:100, Abcam Cat#ab8895), Rabbit anti-H3K4me2 (1:100, Millipore Cat#07-030), Rabbit anti-H3K4me3 (1:100, Active Motif Cat#39159), Rabbit anti-H3K27me3 (1:100, Cell Signaling Tech Cat#9733S). Since pA-MNase does not bind efficiently to many mouse antibodies, we used a rabbit anti-Mouse IgG (1:100, Abcam, Cat#ab46540) as an adapter. H3K27ac was profiled by AutoCUT&RUN in H1 and K562 cells and manually in VUMC-10 and SU-DIPG-XIII cell lines using Rabbit anti-H3K27ac (1:50, Millipore Cat#MABE647). H3K27ac was profiled by AutoCUT&RUN in VUMC-10 and SU-DIPG-XIII cell lines and xenografts using Rabbit anti-H3K27ac (1:100, Abcam Cat#ab45173).

### Cell culture

Human K562 cells were purchased from ATCC (Manassas, VA, Catalog #CCL-243) and cultured according to supplier’s protocol. H1 hESCs were obtained from WiCell (Cat#WA01-lot#WB35186) and cultured in Matrigel™ (Corning) coated plates in mTeSR™1 Basal Media (STEMCELL Technologies cat# 85851) containing mTeSR™1 Supplement (STEMCELL Technologies cat# 85852). Pediatric DMG cell lines VUMC-DIPG-10 (Esther Hulleman, VU University Medical Center, Amsterdam, Netherlands) and SU-DIPG-XIII (Michelle Monje, Stanford University, CA) were obtained with material transfer agreements from the associated institutions. Cells were maintained in NeuroCult NS-A Basal Medium with NS-A Proliferation Supplement (STEMCELL Technologies, cat# 05751), 100 U/mL of penicillin/streptomycin, 20 ng/mL epidermal growth factor (PeproTech, cat# AF-100-15), and 20 ng/mL fibroblast growth factor (PeproTech, cat# 100-18B).

### Patient-derived xenografts

All mouse studies were conducted in accordance with Institute of Animal Care and Use Committee-approved protocols. NSG mice were bred in house and aged to 2–3 months prior to tumor initiation. Intracranial xenografts were established by stereotactic injection of 100,000 cells suspended in 3 μL at a position of 2 mm lateral and 1 mm posterior to lambda. Symptomatic mice were euthanized and their tumors resected for analysis and snap-frozen for storage. To prepare xenograft samples for AutoCUT&RUN, the tissue was thawed and pipetted up-and-down in CUT&RUN wash buffer to break up clumps before adding concanavalin A-coated magnetic beads.

### Annotation and data analysis

We aligned paired-end reads using Bowtie2 version 2.2.5 with options: local—very-sensitive-local—no-unal—no-mixed—no-discordant—phred33 -I 10 -X 700. For mapping spike-in fragments, we also used the—no-overlap—no-dovetail options to avoid cross-mapping of the experimental genome to that of the spike-in DNA [46]. Files were processed using bedtools and UCSC bedGraphToBigWig programs [47, 48].

To examine correlations between the genome-wide distributions of various samples, we generated bins of 500 bp spanning the genome, creating an array with approximately 6 million entries. Reads in each bin were counted, and the log_2_-transformed values of these bin counts were used to determine a Pearson correlation score between different experiments. Hierarchal clustering was then performed on a matrix of the Pearson scores.

To examine the distribution of histone mark profiles around promoters, a reference list of genes for build hg19 were downloaded from the UCSC table browser (https://genome.ucsc.edu/cgi-bin/hgTables) and oriented according to the directionality of gene transcription for further analysis. Genes with TSSs within 1 kb of each other were removed, as were genes mapping to the mitochondrial genome, creating a list of 32,042 TSSs. RNA-sequencing data were obtained from the ENCODE project for H1 and K562 cells (ENCSR537BCG and ENCSR000AEL). RNA reads were counted using featureCounts (http://bioinf.wehi.edu.au/featureCounts/) and converted to Fragments Per Kilobase per Million mapped reads (FPKM) and assigned to the corresponding TSS as a gene expression value. ATAC-sequencing data for H1 cells were obtained from Gene Expression Omnibus (GEO) (GSE85330) and mapped to hg19 using Bowtie2. Mitochondrial DNA accounted for ~ 50% of the ATAC-seq reads and was removed in this study.

All heat maps were generated using DeepTools [49]. All of the data were analyzed using either bash or python. The following packages were used in python: Matplotlib, NumPy, Pandas, Scipy, and Seaborn.

### Training the linear regression model

To ensure the accuracy of fitting histone modification data at promoters to RNA-seq values, genes with more than one promoter were removed from the previously generated TSS list. The genes RPPH1 and RMRP were expressed at extremely high levels in H1 cells and so were considered to be outliers and were removed to avoid skewing the regression, leaving a list of *n* = 12,805 genes.

To assign a relative CUT&RUN signal to promoters for each histone mark, denoted by C, base pair read counts ± 1 kb of the TSS were normalized by both sequencing depth over the promoters being scored and the total number of promoters examined. The prior normalization is to account for both sequencing depth and sensitivity differences among antibodies, and the latter normalization is included so that the model can be applied to different numbers of *cis*-regulatory elements without changing the relative weight of each element. FPKM values were used for RNA-seq.

The linear regression model was trained to fit profiles of histone marks to RNA expression values as previously described [29]. Briefly, we used a linear combination of histone data fitted to the RNA-seq expression values: $y = C_{1} x_{1} + \cdots + C_{n} x_{n}$, where $C_{i}$ is the weight for each histone modification and $x_{i}$ is denoted by $x_{i} = { \ln }\left( {C_{i} +\alpha_{i} } \right)$, where C is the normalized base pair counts described above and $\alpha$ is a pseudo-count to accommodate genes with no expression. The RNA-seq values were similarly transformed as $y_{i} = { \ln }\left( {FPKM_{i} +\alpha_{y,i} } \right)$. Logarithmic transformations were used to linearize the data. A minimization step was then performed to calculate pseudo-counts and weights for each histone modification that would maximize a regression line between CUT&RUN data and RNA-seq.

We expected that the histone marks H3K27ac, H3K27me3, and H3K4me2 would provide the least redundant information. The optimized three histone mark model for K562 cells is described by:

$$\ln \left( {y + 0.0078} \right) = 0.858\ln \left( {C_{\text{H3K27ac}} + 0.058} \right) - 0.615 { \ln }(C_{\text{H3K27me3}} + 0.0816) + 1.609 { \ln }\left( {C_{\text{H3K4me2}} + 0.054} \right).$$

This equation was used to generate all chromatin activity scores.

### Calling chromatin domain for overlap analysis

To compare the global chromatin landscape of H1 and K562 cells chromatin domains were called using a custom script that enriched for regions relative to an IgG CUT&RUN control. Enriched regions among marks were compared and overlaps were identified by using bedtools intersect. Overlapping regions were quantified by the number of common base pairs and these were used to generate the Venn diagrams.

### Venn diagrams

All Venn diagrams were generated using the BaRC webtool, publicly available from the Whitehead Institute (http://barc.wi.mit.edu/tools/venn/).

### Calculating cell-type-specific promoter activity scores

Raw promoter chromatin scores generally fall within a range from − 10 to 10, where a smaller number is indicative of less transcriptional activity. To account for outliers in the data when comparing different cell types, promoter scores within 2 standard deviations were *z*-normalized. Negative and zero values complicate calculating fold change, so the data were shifted in the *x* and *y* directions by the most negative values. The fold difference between promoter scores for various cell types was calculated by dividing the inverse log_10_-normalized promoter scores against each other. A conservative twofold cutoff was used to determine cell-type-specific promoters in each case (Figs. 3b–e, 5a, b). Each list of genes was classified by gene ontology (http://geneontology.org/) to identify statistically enriched biological processes.

To examine the relative similarities between cell types based on their promoter activities, scores for all promoters ≥ 1 kb apart were used to generate an array, and Spearman correlations were calculated for each pair-wise combination of the samples. Hierarchal clustering of the Spearman correlation values was used to visualize the relative similarities between cell types.

### Peak calling on AutoCUT&RUN and ATAC-seq data

Biological replicates profiled by AutoCUT&RUN were highly correlated (Fig. 1b), so replicates were joined prior to calling peaks. The tool MACS2 was used to call peaks [50]. Replicates were joined prior to calling peaks. The tool MACS2 was used to call peaks, and the following command was used on the command line: “macs2 callpeak -t file -f BEDPE -n name -q 0.01 –keep-dup all -g 3.137e9.” An FDR cutoff of 0.01 was used.

### Calculating cell-type-specific enhancer activity scores

To assemble a list of distal *cis*-regulatory elements in the human genome, we used MACS2 to call peaks on H3K4me2 profiles from each of our samples using the same flags described in the “Peak calling on AutoCUT&RUN and ATAC-seq” methods section. To distinguish between TSSs and putative enhancers, peaks < 2.5 kb away from an annotated TSS were removed, and windows ± 1 kb around these putative enhancers were assigned chromatin activity scores using the algorithm trained to predict promoter activity. Correlation matrices comparing the enhancer scores between samples were generated in the same manner as the correlation matrix comparing promoter scores between samples.

### Data access

All data generated and used in this manuscript have been deposited in GEO: GSE120011.

## Additional files

**Additional file 1.** Supplementary figures.

**Additional file 2.** Supplementary table.
