# Supplementary material for: Automated in situ chromatin profiling efficiently resolves cell types and gene regulatory programs
Source: Epigenetics Chromatin. 2018 Dec 21;11:74. doi: 10.1186/s13072-018-0243-8 (PMC6302505; doi:10.1186/s13072-018-0243-8)
Supplement: Supplementary file 1 — Additional file 1. Supplementary figures. [file 13072_2018_243_MOESM1_ESM.pdf]

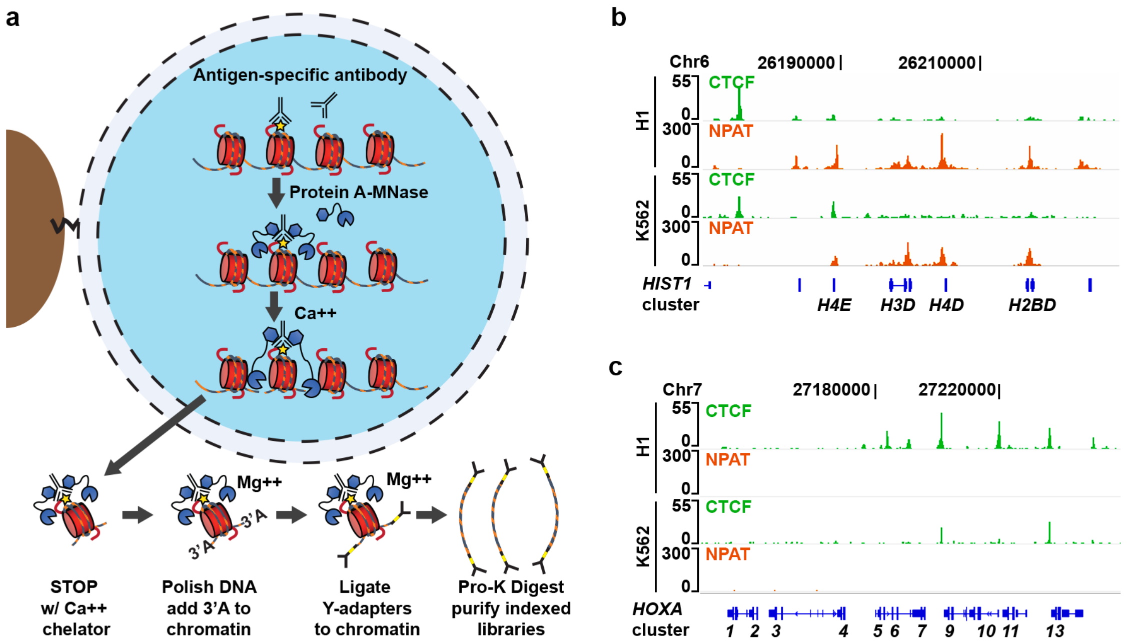

Additional file 1: Fig. S1

AutoCUT&RUN accurately maps NPAT and CTCF. **a** A modified CUT&RUN protocol allows for automation. ConA bead-bound samples are incubated with a chromatin protein-specific antibody, and arrayed on the Biomek for successive washes, tethering of a proteinA-MNase fusion protein, and cleavage of DNA by adding Ca^{2+}. To avoid purifying the digested DNA prior to library prep, the reaction is stopped with an EGTA only STOP buffer which specifically chelates Ca^{2+} while leaving adequate Mg^{2+} to allow End-polishing and Ligation of Illumina Y-adapters to the chromatin fragments. Chromatin protein is then digest with Proteinase-K and the indexed CUT&RUN libraries are purified on the Biomek using Ampure Magnetic Beads. **b** Genome browser tracks of NPAT and CTCF AutoCUT&RUN showing NPAT enrichment at promoters of the *HIST1* gene cluster in both H1 and K562 cells. **c** Genome browser tracks confirming CTCF is bound to insulator regions in the *HOXA* locus.

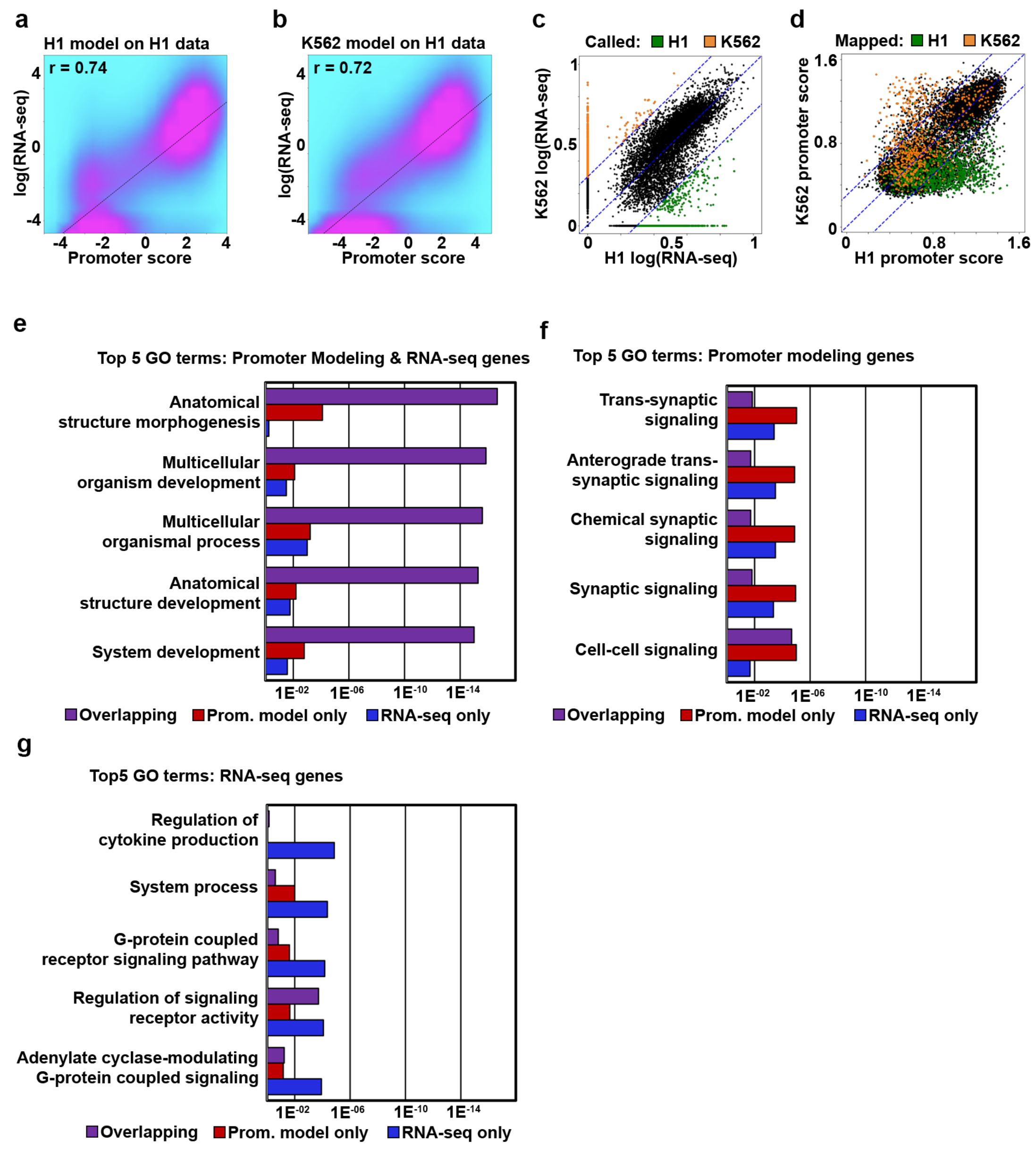

Additional file 1: Fig. S2

Developing a linear regression model to predict the activity of *cis*-regulatory elements. **a** Density scatterplot comparing H1 RNA-seq values for single-promoter genes to H1 promoter scores predicted by the model trained on H1 data. **b** Density scatterplot comparing H1 RNA-seq values for single-promoter genes to H1 promoter scores predicted by the model trained on K562 data. **c**

Scatterplot of RNA-seq values for single-promoter genes in H1 and K562 cells. Colored dots indicate the RNA expression levels are $\geq$2-fold enriched in either H1 cells (green) or K562 cells (orange). **d** Scatterplot showing the distribution of genes with RNA-seq values that are $\geq$2-fold enriched in either H1 cells (green) or K562 cells (orange) mapped onto their corresponding promoter chromatin scores. Blue dotted lines indicated the 2-fold difference cut-off. **e** Bar graph showing the top five Gene Ontology (GO) terms overrepresented in the collection of cell-type specific genes identified by both promoter activity modeling as well as RNA-seq (purple), and the relative enrichment of these terms in the collections of genes uniquely identified as cell-type specific by promoter activity modeling (red) or RNA-seq (blue). **f** Bar graph showing the top five GO terms overrepresented in the collection of genes identified as cell-type specific according to promoter modeling scores only. **g** Bar graph showing the top five GO terms overrepresented in the collection of genes uniquely identified as cell-type specific according to RNA-seq.

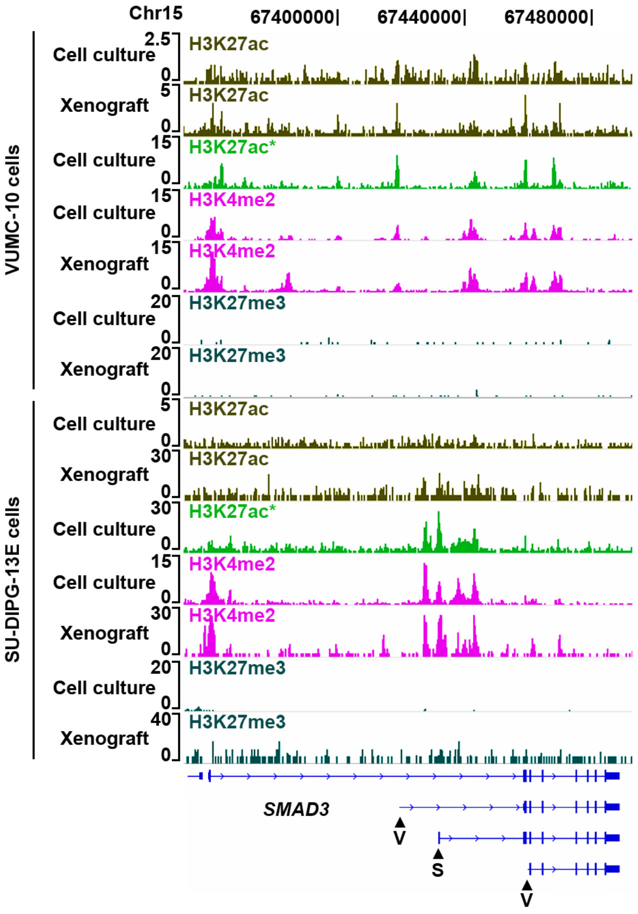

Additional file 1: Fig. S3

DMG subtype-specific *SMAD3* promoter activities. Genome browser tracks of histone marks profiled by AutoCUT&RUN in VUMC-10 and SU-DIPG-XIII cells at a representative locus (*SMAD3*) showing the concordance of profiles from cell culture and xenograft samples. The H3K27ac signal in SU-DIPG-XIII cells was noisy, but this issue is antibody specific. For comparison H3K27ac was also profiled manually using an alternative antibody (*). Arrowheads indicate promoters that are predicted to be specifically active in VUMC-10 (V) or SU-DIPG-XIII (S) cells.

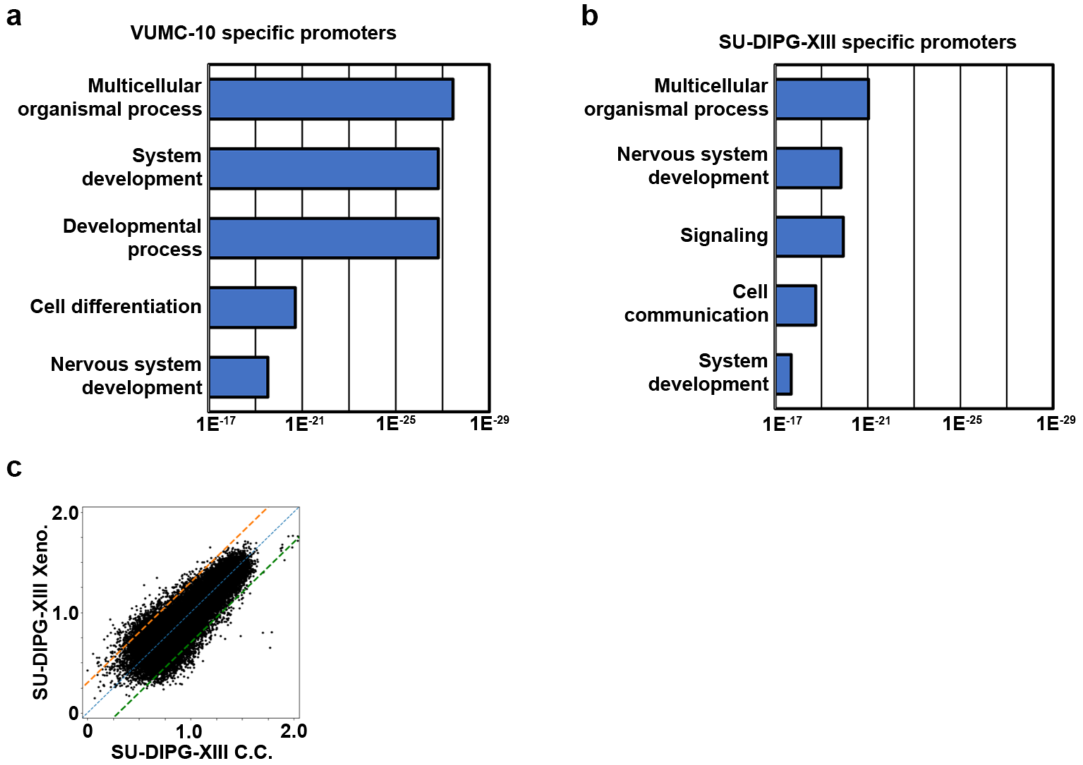

Additional file 1: Fig. S4

AutoCUT&RUN identifies DMG specific gene regulatory programs. **a** GO terms that are overrepresented in the collection of promoters that are $\geq$2-fold enriched in VUMC-10 cells according to promoter chromatin scores. **b** GO terms that are overrepresented in the collection of promoters that are $\geq$2-fold enriched in SU-DIPG-XIII cells according to promoter chromatin scores. **c** Scatterplot comparing the promoter scores of SU-DIPG-XIII cell culture (C.C.) and xenograft (Xeno.) samples. 1,619 promoters have a $\geq$2-fold difference in promoter chromatin scores between these samples.

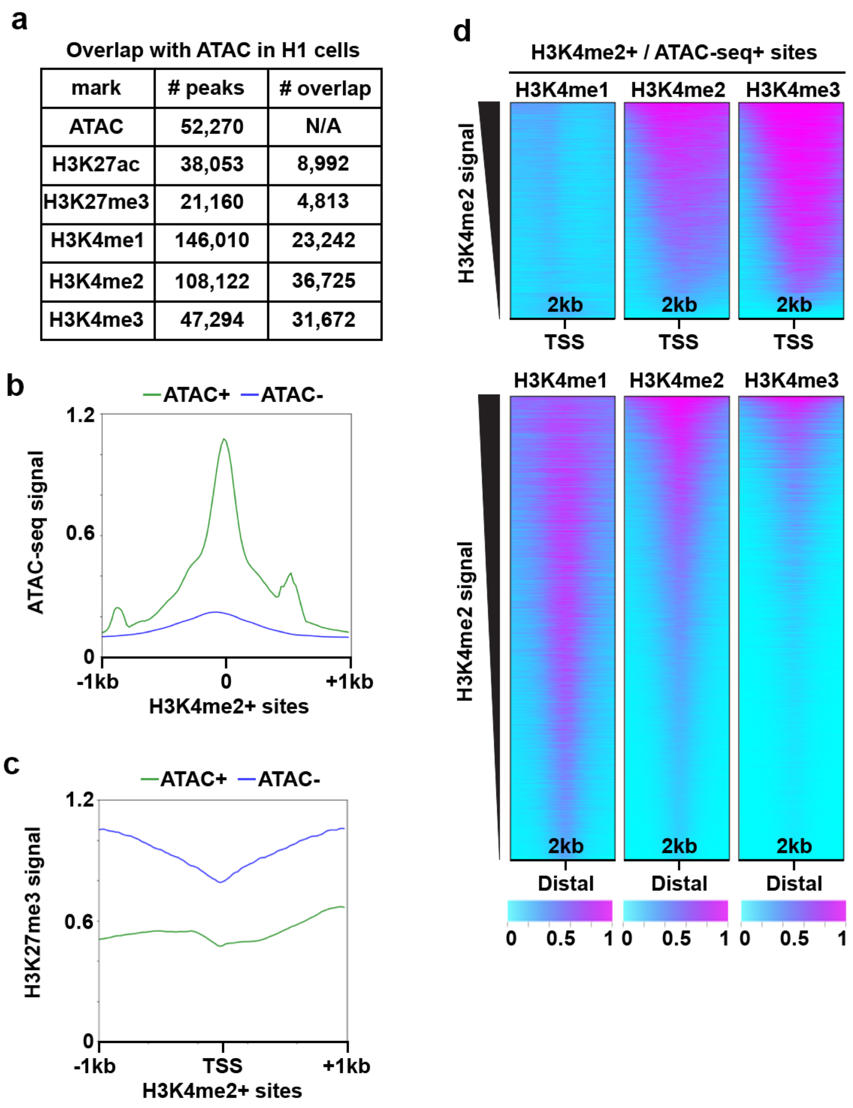

mark	# peaks	# overlap
ATAC	52,270	N/A
H3K27ac	38,053	8,992
H3K27me3	21,160	4,813
H3K4me1	146,010	23,242
H3K4me2	108,122	36,725
H3K4me3	47,294	31,672

Additional file 1: Fig. S5

AutoCUT&RUN is a sensitive method to distinguish proximal and distal *cis*-regulatory elements. **a** Table of the overlap of accessible chromatin sites (ATAC-seq peaks) and peaks called on various AutoCUT&RUN profiles of histone marks in H1 cells. **b** Mean enrichment of ATAC signal at H3K4me2 peaks that were either called as ATAC+ (green) or ATAC- (blue). **c** Mean enrichment of H3K27me3 signal at H3K4me2+ TSSs that were either called as ATAC+ (green) or ATAC- (blue). **d** Heat maps showing the distribution of normalized H3K4me1,

H3K4me2 and H3K4me3 profiles over all H3K4me2+/ATAC+ TSSs and distal regulatory elements (Distal).
